# Deoxyribonucleic acid methylation profiling of single human blastocysts by methylated CpG-island amplification coupled with CpG-island microarray

**DOI:** 10.1016/j.fertnstert.2015.03.020

**Published:** 2015-06

**Authors:** John Huntriss, Karen Hemmings, Praveen Baskaran, Lee Hazelwood, Kay Elder, Carl Virtanen, David Miller, Helen M. Picton

**Affiliations:** a Division of Reproduction and Early Development, Leeds Institute of Cardiovascular and Metabolic Medicine, University of Leeds, Leeds, United Kingdom; b School of Molecular and Cellular Biology, Faculty of Biological Sciences, University of Leeds, Leeds, United Kingdom; c Bourn Hall Clinic, Cambridge, United Kingdom; d Princess Margaret Cancer Centre, Toronto, Ontario, Canada

**Keywords:** Preimplantation, CpG island, methylation, blastocyst, epigenetic

## Abstract

**Objective:**

To study whether methylated CpG-island (CGI) amplification coupled with microarray (MCAM) can be used to generate DNA (deoxyribonucleic acid) methylation profiles from single human blastocysts.

**Design:**

A pilot microarray study with methylated CpG-island amplification applied to human blastocyst genomic DNA and hybridized on CpG-island microarrays.

**Setting:**

University research laboratory.

**Patient(s):**

Five cryopreserved sibling 2-pronuclear zygotes that were surplus to requirements for clinical treatment by in vitro fertilization were donated with informed consent from a patient attending Bourn Hall Clinic, Cambridge, United Kingdom.

**Intervention(s):**

None.

**Main Outcome Measure(s):**

Successful generation of genome-wide DNA methylation profiles at CpG islands from individual human blastocysts, with common genomic regions of DNA methylation identified between embryos.

**Result(s):**

Between 472 and 734 CpG islands were methylated in each blastocyst, with 121 CpG islands being commonly methylated in all 5 blastocysts. A further 159 CGIs were commonly methylated in 4 of the 5 tested blastocysts. Methylation was observed at a number of CGIs within imprinted-gene, differentially methylated regions (DMRs), including placental and preimplantation-specific DMRs.

**Conclusion(s):**

The MCAM method is capable of providing comprehensive DNA methylation data in individual human blastocysts.

**Discuss:** You can discuss this article with its authors and with other ASRM members at **http://fertstertforum.com/huntrissj-cpg-island-amplification-human-blastocysts/**

Epigenetic programming, which is essential for normal development, is highly regulated during mammalian gametogenesis and preimplantation development (1) and includes the modification of a range of DNA (deoxyribonucleic acid) sequence elements by DNA methylation (2, 3). The epigenetic programming of human gametes and preimplantation embryos that develop in vitro may be affected by assisted reproductive technology (ART) (4). Moreover, ART seems to induce subtle epigenetic effects that may lead to greater risk of diseases in adult life (5–9). In human ART, it is imperative to provide a regimen that supports epigenetic programming that is compatible, as far as possible, with normal development of the conceptus. Defining the DNA methylome of the human preimplantation embryo may reveal which genomic regions may be susceptible to the effects of embryonic development in vitro, by identifying their requirement for a specific methylation state in early development.

In this pilot study, we sought to identify whether genome-wide DNA methylation analysis of CpG (C–phosphate–G) islands is feasible in single human blastocysts. These islands (CGIs) are regions of the genome important in regulating gene expression in a manner dictated by their methylation status (10, 11). Methylated CGI amplification coupled with microarray (MCAM) (12) is a method of DNA methylation analysis that has been used to assess CGI methylation in several human disease states (13–15).

## Materials and methods

### Samples

In the current report, a cohort of 5 cryopreserved sibling in vitro fertilization (IVF), 2-pronuclear zygotes (surplus to requirements for clinical treatment) were donated, with informed consent, from a couple attending Bourn Hall Clinic (Cambridge, United Kingdom) (maternal age: 29 years). Embryos were obtained under protocols approved by the local research ethics committees, which were licensed by the Human Fertilisation and Embryology Authority. Zygotes were thawed, equilibrated, and cultured individually to the blastocyst stage under embryo-tested mineral oil at 37°C under 5% CO_2_ in humidified air.

**Supplemental Figure 1:**
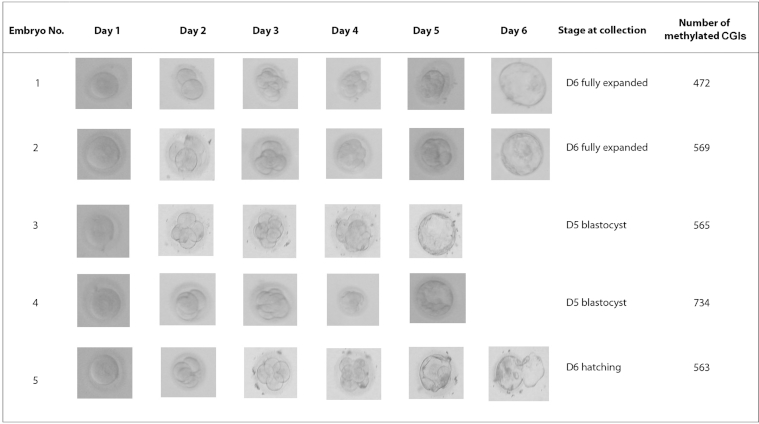
Images of the human preimplantation embryos used in the MCAM study, indicating the number of methylated CGIs in each embryo. CGI = CpG island; MCAM = methylated CpG island amplification coupled with microarray.

Embryos numbered 1, 3, and 5 were cultured in 4-μl droplets of a defined embryo culture medium based on the composition of human tubal fluid that was comprised of Earle's balanced salt solution, supplemented with 1 mmol/L of glucose, 5 mmol/L of lactate, 0.47 mmol/L of pyruvate, 0.5% (vol/vol) human serum albumin (Zenalb 20, Bio Products Laboratory), and amino acids at close-to-physiologic concentrations, essentially as previously described (16–18). Embryos numbered 2 and 4 were initially cultured in 4-μl droplets of EmbryoAssist before transfer to equivalent-sized microdrops of BlastAssist medium (both from Origio). Morphologic grading was recorded as described previously (18). The blastocysts used in the study are shown in Supplemental Figure 1 (available online).

At the end of culture, blastocysts were allowed to perish. They were washed in Ca^2+^ and *Mg*^*2+*^-free phosphate buffered saline (Life Technologies, Ltd) at 4°C. Zona pellucidae were removed by brief exposure to acid Tyrode's solution (Sigma), and each individual blastocyst was snap-frozen in lysis buffer (Dynal, Life Technologies, Ltd). Genomic DNA was isolated from blastocysts using the Qiagen DNA Micro kit (Qiagen).

### Methylated CpG-Island Amplification Coupled with CpG-Island Microarray

Methylated CGI amplification was performed according to earlier reports (12, 15), with minor modifications. Briefly, embryonic DNA was digested for 16 hours in total, with 2 consecutive digestions of 8 hours, each using 8 units of methylation-sensitive *SmaI* restriction enzyme (New England Biolabs) at 25°C, followed by heat inactivation (95°C, for 10 minutes). Subsequently, DNA was digested with 15 units of *XmaI* for 6 hours, at 37°C, followed by heat inactivation.

The residual methylated DNA fragments were precipitated with alcohol and ligated to 0.5 nmol of RXMA polymerase chain reaction (PCR) adaptors (12), using T4 DNA ligase (New England Biolabs) in a 10-μl reaction volume. The RXMA PCR adaptors were prepared by combining the 2 oligonucleotides RXMA24 (5-AGCACTCTCCAGCCTCTCACCGAC-3) and RXMA12 (5-CCGGGTCGGTGA-3) at 65°C for 5 minutes, and annealed by cooling.

Methylated DNA was amplified by PCR using the entire ligation reaction in a 50-μL volume containing 100 pmol of RXMA24 oligonucleotide, and 5 units of Advantage 2 Taq DNA polymerase (Clontech). Before amplification, the PCR reaction mixture was incubated at 72°C for 5 minutes, and 95°C for 3 minutes. The DNAs were subjected to 35 cycles of 1 minute at 95°C, and 3 minutes at 72°C, with a final extension step (72°C) of 10 minutes.

A 5-μl aliquot of each PCR product was assessed on a 1.2% (w/v) agarose gel, with ethidium bromide staining, with a smear from 300 bp to 3 Kb indicating successful amplification of methylated DNA. The MCAM PCR products from each blastocyst were purified using the PCR purification kit (Qiagen), and labelled and hybridized individually to Agilent 244K Human CpG island arrays that feature 27,800 CGIs, at the Ontario Cancer Institute Genomics Centre. Bioinformatic processing and subsequent bioinformatic analysis was performed, as detailed in the Supplemental Bioinformatic Methods (available online). Blastocyst methylation data were compared against methylation data obtained from the ENCODE project for cell lines including GM12878, HI-hESC (human embryonic stem cell), HeLa-S3, HUVEC, K562, HMEC, and HepG2 (see Supplemental Bioinformatic Methods).

## Results

Methylated CGI amplification was successful for each of the 5 blastocysts. After hybridization of these products individually to Agilent 244K Human CpG island arrays, data analysis identified 2,903 methylated CGIs in total, across all 5 blastocysts (see Supplemental Bioinformatic Methods for further details). Of these, 1,263 CGIs were methylated in ≥1 blastocyst (data available upon request). Thus, for embryos 1–5, respectively, the number of methylated CGIs was 472, 569, 565, 734, and 563.

**Figure 1:**
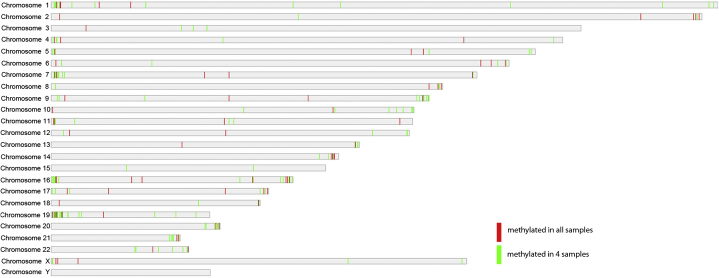
Distribution of CpG islands that are methylated in blastocysts across the human genome, as generated by MCAM. *Red bars* represent CGIs methylated in all 5 embryos; *green bars* represent CGIs methylated in 4 of 5 embryos. CGI = CpG island; CpG = C phosphate G; MCAM = methylated CpG island amplification coupled with microarray.

**Figure 2:**
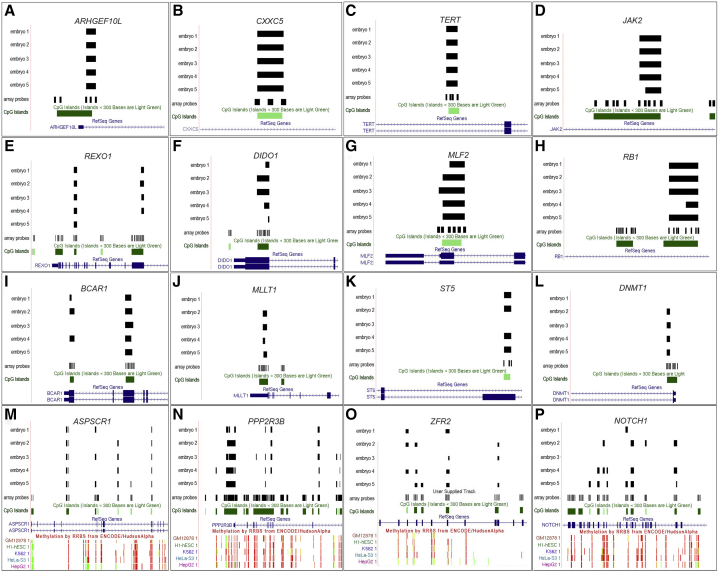
(**A–H**) Examples of loci with CpG islands that are methylated in all embryos (5 of 5), as obtained from the University of California, Santa Cruz browser. *Black horizontal bars* represent methylation coverage across a defined CGI (*green*) for each embryo. Overlapping loci (RefSeq genes) are *blue*. (**I–L**) Examples of loci with CpG islands that are methylated in 4 of the 5 tested embryos. (**M–P**) Examples of loci with multiple contiguous methylated CpG islands in most embryos. For these panels, the corresponding methylation data, as obtained from the ENCODE data for the human cells lines GM12878, HI-hESC (human embryonic stem cell), K562, HeLa-S3, and HepG2 are shown.

We hypothesized that the most stringent data would be represented by CGIs that were methylated in all 5 blastocysts, and observed 121 CGIs that met this criterion (24 CGIs were expected by chance; see Supplemental Table 1, available online). The distribution of the methylated CGIs across the genome is shown in Figure 1, with CGIs that were methylated in all 5 blastocysts listed in Supplemental Table 2 (available online). Specific examples of CGIs methylated in all 5 blastocysts are shown in Figure 2A–2H.

**Supplemental Figure 2:**
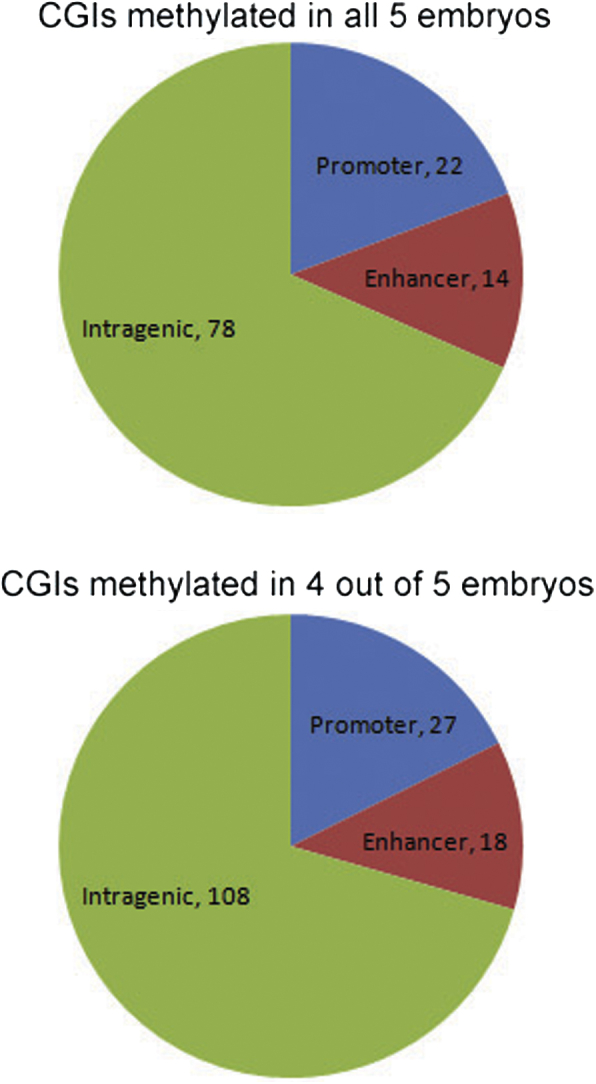
Analysis of the CGIs that were methylated in ≥4 embryos, to identify whether the methylated embryonic CGIs overlap with gene promoters, enhancers, or intragenic regions. The analysis of promoter, enhancer, and intragenic overlaps were performed independently; therefore, in some cases, the same CGI might overlap enhancer/promoter and intragenic regions, and therefore may have been counted more than once. CGI = CpG island.

The methylated CGIs detected by MCAM included those that were located toward the subtelomeric regions, consistent with observations of chromosomal centric and pericentric hypomethylation in mouse preimplantation embryos (19), and subtelomeric hypermethylation in human induced pluripotent cells (20). We observed 159 CGIs that were methylated in 4 of the 5 tested blastocysts (see Fig. 2I–2O; and Supplemental Table 3, available online). The *NOTCH1* gene had 2 CGIs that were methylated in 3 of the 5 embryos, and several additional CGIs that exhibited variable methylation between embryos (Fig. 2P). The distribution of methylated CGIs, within gene promoters, enhancers, and intragenic regions, are shown in Supplemental Figure 2 (available online) and reveals a predominance of CGI methylation in the gene body.

**Supplemental Figure 3:**
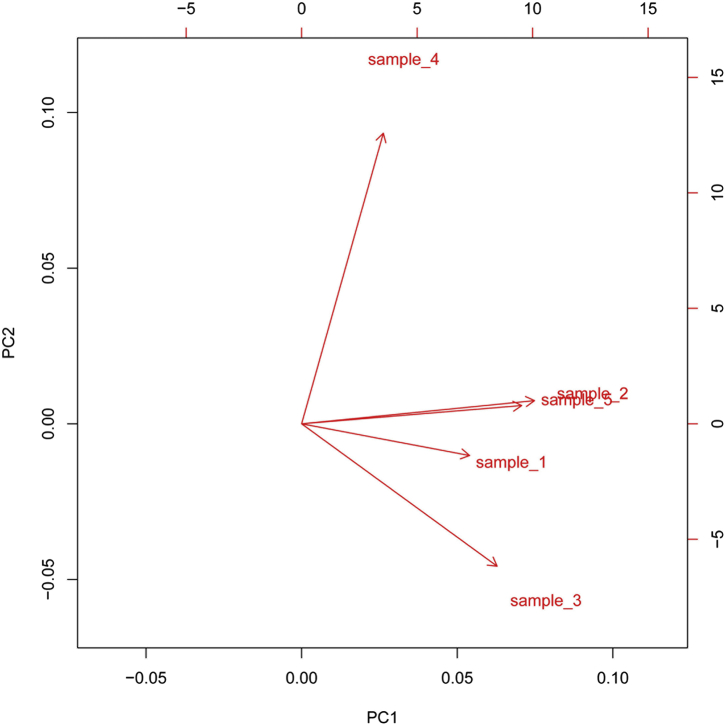
Principal component analysis and hierarchical clustering show that sample 4 was the most divergent, relative to the other 4 samples. PC = principal component.

**Supplemental Figure 4:**
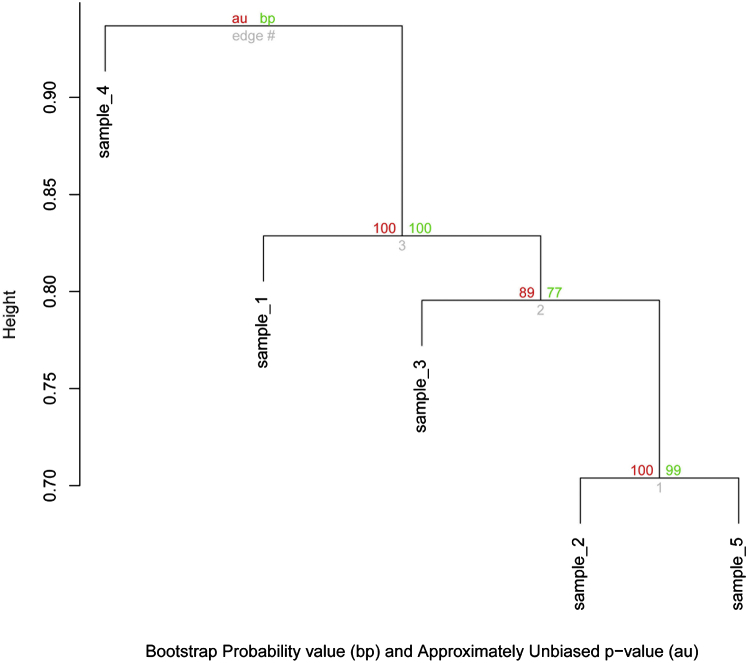
Cluster dendrogram of methylation pattern with bootstrap resampling.

Gene ontology analysis was undertaken to investigate whether the methylated loci possess a common ontology term, and identified chromatin remodelling complex, negative regulation of transcription, transcriptional repressor complex, and negative regulation of the RNA (ribonucleic acid) metabolic process as the significantly over-represented terms (Supplemental Table 4, available online). Functional annotations of the genes with methylated CGIs in all blastocysts identified annotations including proto-oncogene, glioma, small cell lung cancer, and extracellular matrix–receptor interaction categories (Supplemental Table 5, available online). Principal component analysis and hierarchic clustering were performed using the 1,263 positions that were methylated in ≥1 sample (see Supplemental Figs. 3 and 4, available online). Both of these methods show that sample 4 was the most divergent, relative to the other 4 samples.

Methylation of DNA was observed in blastocysts at CGIs that were located within or proximal to 27 transcripts that are known or predicted to be imprinted (Supplemental Table 6, available online), although not all of these CGIs were methylated in every blastocyst. Methylation was observed in at least 15 known imprinted gene differentially methylated regions (DMRs), including several that are specific to the placenta and/or preimplantation embryos.

## Discussion

In this study, we established that the MCAM method is suitable for CGI methylation analysis in individual human blastocysts. We observed 121 CGIs that were methylated in all tested embryos, indicating that these regions may represent an epigenetic pattern common to this stage of human development.

Methylation was observed at 27 known or predicted imprinted genes, including at least 15 known imprinted gene DMRs (Supplemental Table 6). A conclusive determination of whether the methylated DMRs identified by MCAM represented methylation imprints was not possible, because methylation imprints are expected to be in the region of 35% to 65% methylation (21). The MCAM protocol as applied in this study would be likely to identify only the DMRs with the higher levels within that range. We suggest, therefore, that quantitative approaches, such as pyrosequencing methylation analysis, be applied in future experiments, to precisely determine the methylation status of these regions.

An additional consideration is that some of the methylation observed at imprinted genes may not be relevant to the process of imprinting—for example, in areas where methylation occurs outside of known DMRs. Among the CGIs methylated in all 5 blastocysts was CpG island 85, which corresponds to the DMR of the imprinted transcript of the RB1 gene (Fig. 2H), a DMR that we previously demonstrated, using pyrosequencing, to be methylated in human blastocysts (22). In addition, methylation was observed at known DMRs for the imprinted GNAS locus transcripts on chromosome 20.

Methylation was observed in 5 known placental and/or preimplantation-specific DMRs (Supplemental Table 6) that are associated with the imprinted transcripts *GLIS3*, *AIFM2, FAM196A/DOCK1, DNMT1* (see Fig. 2L), and *RHOBTB3*. However, not all the embryos were methylated at these regions. Therefore, one possibility is that some placental-specific methylation imprints initially become detectable in the human blastocyst, possibly in the trophectoderm, although further experimentation is required to clarify this. We suggest that CpG 121 within the neurotrimin gene (*NTM)* may represent a placental-specific DMR that is also detectable in late preimplantation development; therefore, further investigation is required for confirmation.

For some loci, multiple contiguous CGIs were shown to be methylated in each embryo, although methylation varied, both in extent and specific CGI marking across such regions between embryos (for example, *ASPSCR1, PLXNB2, PPP2R3B, ZFR2,* and *NOTCH1*; see Fig. 1M–1P). The observed differences in DNA methylation between embryos may be due to interembryo variability in developmental competence, growth, and metabolism, although further research is required to explore these possible influences. We found that 88 of the 121 blastocyst-methylated CGIs were additionally methylated in other cell lines as defined by the ENCODE project. This finding suggests that the remaining sites may include some blastocyst-specific methylated sites, (indicated as “not confirmed” in Supplemental Table 2). Given that these CGIs were not methylated in the HI-hESC human embryonic stem cell line, some of the methylated CGIs that were identified in blastocysts may include trophectoderm-specific methylation sites; however, further experiments are required to confirm this possibility.

In some cases, ≥2 CpG islands within a particular locus were observed to be methylated. The genes *KIF26A*, *GAS6*, *CACNA1H*, *TAOK2*, *CTDP1,* and *BSG* were observed to have 2 methylated CGIs in all 5 blastocysts, and all 5 embryos exhibited 3 methylated CGIs at *ASPSCR1*. The genes *SLC9A3*, *SLC12A7*, *MAD1L1*, *IQCE*, *PTPRN2*, *CACNA1B*, *LRP5*, *PPP2R3B,* and *PLEC* were all observed to have 2 methylated CGIs in 4 of 5 blastocysts.

The number of highly methylated CGIs in human blastocysts is likely to be low relative to gametes and other tissues, owing to the prior genome-wide erasure of methylation during earlier preimplantation development, as confirmed by the low methylation levels reported in mouse blastocysts (23, 24). However, some of the methylated CGIs detected in our study may have survived erasure during preimplantation development or may be the product of the initial stages of de novo methylation, corresponding well with the expression of DNA methyltransferases in human blastocysts (25, 26).

We acknowledge that our pilot MCAM study is limited in that only 5 individual blastocysts were analyzed. These single-embryo hybridizations to CpG island arrays have identified only the most highly methylated CGIs that were consistent across the tested embryos. Cohybridization experiments (2-color) against other suitable cell types are highly likely to reveal additional developmental stage–specific methylation data, and regions of hypomethylation in human blastocysts. Unfortunately, further embryonic DNA was not available to perform these experiments.

In addition, we acknowledge that quantitative, higher-resolution methods of DNA methylation analysis, such as reduced representation bisulphite sequencing, have been used very successfully for DNA methylation profiling in mouse oocytes and preimplantation embryos (23, 24, 27), but these earlier studies required the pooling of large numbers of embryos, a strategy that cannot be applied for the study of human preimplantation embryos. More recently, however, the sensitivity of this type of sequencing has been improved, to allow comprehensive DNA methylation analysis in pooled samples containing small numbers of embryos (28–30).

The MCAM analysis reported here permitted the analysis of individual embryos to reveal that although methylation patterns among embryos vary considerably, a common set of CGIs are methylated in all (5 of 5) or most (4 of 5) embryos. Such findings would be masked by pooling samples before analysis. Therefore, MCAM may be useful as a method for methylation profiling of CpG islands in individual or small numbers of pooled preimplantation embryos. A number of cancer-related genes (e.g., *RB1*, *ST5*, *MTA1*, *CXXC5*, *GAS6*, *SMARCA4, JAK2, WNK2,* and *HDAC4*) were shown in this study to have methylated CGIs.

This pilot study did not attempt to evaluate the impact of various culture media conditions on CGI methylation in human blastocysts, because the numbers of sibling embryos available for analysis within this cohort were far too few to support such a challenging agenda. However, the 121 commonly methylated CGIs were conserved between embryos cultured in 2 different culture systems, regardless of whether a single medium (EBSS with defined supplements) or a sequential system (EmbryoAssist followed by BlastAssist) was used.

Further, when principal component analysis and hierarchic clustering were performed using the 1,263 CGIs that were methylated in ≥1 of the blastocyst samples (Supplemental Figs. 3 and 4), both methods of analysis indicated that similar methylation patterns were observed, especially between blastocysts 2 and 5, suggesting consistency between blastocysts derived after culturing in 2 different media systems. However, the same methods indicated that methylation of blastocyst 4 (cultured in EmbryoAssist followed by BlastAssist) was distinct from the other 4 blastocysts analyzed. The methylation differences observed for this particular embryo may, however, involve several factors that cannot be elucidated in the present study.

A method similar to MCAM has been used to assess DNA methylation on chromosome 7 in single mouse blastocysts (31). Use of this method supports our conclusions that methods that employ methylation-sensitive enzyme digestion, when coupled with microarrays, provide a viable approach for genome-wide DNA methylation analysis when single-embryo analysis is required.

## Supplementary data

Supplemental Bioinformatic Methods
