# Supplementary material for: Deoxyribonucleic acid methylation profiling of single human blastocysts by methylated CpG-island amplification coupled with CpG-island microarray
Source: Fertil Steril. 2015 Jun;103(6):1566–1571.e4. doi: 10.1016/j.fertnstert.2015.03.020 (PMC4449363; doi:10.1016/j.fertnstert.2015.03.020)
Supplement: Supplemental Bioinformatic Methods [file mmc1.docx]

**Supplemental Bioinformatic Methods**

DNA Methylation Profiling of Single Human Blastocysts by Methylated CpG Island Amplification Coupled with CpG-island Microarray (MCAM).

**Data processing**

The Ontario Cancer Institute Genomics Centre (Toronto) carried out initial data processing of the Agilent microarrays**.** Array data were checked for overall quality using R (v2.10.0) and the Agilent Feature Extraction Software (v10.7.3.1). All samples were deemed of suitable quality for further analysis. Data was extracted and compiled using PERL scripts and for any probes that were duplicated, the mean of their signals was taken as the final value. The probes locations in the genome were taken as the mid-point between the start and end positions. Probes were then sorted and output in ascending base pair order for each chromosome. Data analysis and normalization was accomplished using CisGenome (1) which is based on algorithms found in TileMap v2.0 (2). The corresponding Tilemap command line tools were also used. Normalization and log_2_ transformation was performed in TileMap and follows basic quantile style normalization (3). Parameters in the configuration file were changed to account for the 60bp length of probes and 100bp average spacing of probes within the CpG islands that are tiled on this array platform. The moving average (MA) algorithm was used to find peaks with a maximal test statistic of 3.0 as a lower bound cut-off. Results were output into BED file format for further processing.

The BHRC Bioinformatics technology group (University of Leeds, UK) has performed the following data analysis.

**Statistical Analysis**

The processed data (in BED file format) for each blastocyst were parsed, sorted and merged using BEDTools (4) to identify 1263 methylated genomic CGIs across the five tested blastocysts (data available upon request). 121 CGIs were methylated in all 5 tested embryos whilst 159 CGIs were methylated in four out of five blastocysts. The significance of these sites has been assessed using a Chi square test (with Yate’s continuity correction), see Supplemental Table 1). The probability of a site being methylated is (the number of methylation sites in sample) **÷** (total number of methylation sites). We consider the total number of methylation sites to be either 1263 (those methylated in our samples) or 27800 (the total number in the array). The former provides a more stringent criterion to assess the significance of sample sites, though a value in-between may be more appropriate in reality. The data shows that the number of CGIs methylated in all five embryos (121) is significantly higher than the number expected by chance, when taking 1263 as the total number of possible sites. Data for CGIs that were methylated in four out of five embryos were not found to be significantly different from expected, when taking 1263 as the total number of possible sites. However, taking the total number of possible sites as 27800, leads to all sites being significantly higher than the expected value. The genomic positions of the 121 sites methylated in all five blastocysts can be found in Supplemental Table 2. The genomic positions of the 159 sites methylated in four out of the five tested blastocysts can be found in Supplemental Table 3.

**Promoter and Enhancer Analysis:**

We have presented the number of the methylated CGIs located within promoters, enhancers and intragenic regions (Supplemental Figure 2). The methylation marks are defined to fall in a region (e.g. promoter) provided they overlap with the same region as determined in at least 5 out of the following human cell lines NHLF, NHEK, HUVEC, HSMM, HMEC, HEPG2, K562, H1-hESC AND GM12878 mapped to HG19 (5).

**Functional similarity of methylated regions:**

In order to investigate whether there was any common functional significance between methylated genes, we performed Gene Ontology and Pathway analysis using Cytoscape (6) and the BiNGO plugin (7). Gene ontology analysis and functional annotation of these genes was performed in Cytoscape and DAVID (The Database for Annotation, Visualization and Integrated Discovery) (8). Genes that corresponded to the methylated positions were retrieved for the CGIs that were methylated in all five samples using BioMart R package (9,10). Overrepresented terms included chromatin remodelling complexes, transcriptional repressor complex, and negative regulation of binding. The complete ontology and the DAVID pathway analysis is shown in Supplemental Table 4 and Supplemental Table 5.

**Comparison with other methylation patterns:**

Methylation data was visualized in the UCSC browser (11,12). We also wished to investigate the novelty of the observed methylation signatures by comparing the 121 sites to methylation observed in other human cell types cells. Methylation data is available through the ENCODE project for cell lines GM12878, HI-hESC (human embryonic stem cell), HeLa-S3, HUVEC, K562, HMEC and HepG2 cell lines produced by HudsonAlpha Institute for Biotechnology (13). We found that 88 out of the 121 blastocyst methylated CGIs were also methylated in these cell lines. This result provides us with confidence in our experimental protocol and also suggests that the data includes some potentially novel blastocyst-specific methylated sites, (indicated as ‘not confirmed’ in Supplemental Table 2, right hand column), possibly including trophectoderm-specific sites. Further experiments are required to confirm this.

**Repetitive elements analysis:**

Using the probe location data for the Agilent array (available from <http://www.chem.agilent.com/cag/bsp/gene_lists.asp>) we performed overlapping analysis against repetitive element locations (available within the UCSC genome browser repeat masker data (14). We found that there were 132410 probes out of all probes (237221) to be common, that is, 56% of the data. In general, we found that these regions were under-represented in our methylation data. For example, locations where CGI methylation was present in all five blastocyst samples, only 8 out of the 121 CGIs were in repetitive elements (including 1 LINE, 1 LTR, 5 simple repeats and 1 GC-rich low complexity region). A similar result was observed for the CGIs that were methylated in 4 out of the 5 blastocyst samples, with 13 out of the 159 methylated CGIs being found in repetitive elements that included 1 SINE, 6 simple repeats, 1 tandemly repeated ribosomal RNA (rRNA) element and 5 GC-rich low complexity regions. This was again repeated in 3 or greater samples, with 59 out of the 983 in repetitive elements (6 LINE, 3 LTR, 3 SINE, 7 simple repeats and 40 low complexity regions).

**Principal Component Analysis (PCA) and hierarchical clustering of methylated CGIs:**

Principal Component Analysis (PCA) and hierarchical  clustering using the 1263 positions that were methylated in one or more samples shows that Sample 2 and Sample 5 are clustered together, indicating that these embryos demonstrated similar methylation pattern (see Supplemental Figures 3 and 4). The same assessments however separated sample 4 from the rest.

**References**

1) Hongkai Ji, Hui Jiang, Wenxiu Ma, David S. Johnson, Richard M. Myers and Wing H. Wong. An integrated software system for analyzing ChIP-chip and ChIP-seq data. Nature Biotechnology 2008; 26: 1293-1300.

2) Hongkai Ji and Wing H. Wong. TileMap: create chromosomal map of tiling array hybridizations. Bioinformatics 2005; 21: 3629-3636

3) Bolstad BM, Irizarry RA, Astrand M, Speed TP. A comparison of normalization methods for high density oligonucleotide array data based on variance and bias. Bioinformatics. 2003;19:185-9

4) Quinlan AR and Hall IM, 2010. BEDTools: a flexible suite of utilities for comparing genomic features. Bioinformatics. 2010; 26: 841–842.

5) Ernst J, Kheradpour P, Mikkelsen TS, Shoresh N, Ward LD, Epstein CB, Zhang X, Wang L, Issner R, Coyne M, et al. Mapping and analysis of chromatin state dynamics in nine human cell types.  Nature 2011; 473:43-49.

6) Cline MS, Smoot M, Cerami E, Kuchinsky A, Landys N, Workman C, Christmas R, Avila-Campilo I, Creech M, Gross B, Hanspers K, Isserlin R, Kelley R, Killcoyne S, Lotia S, Maere S, Morris J, Ono K, Pavlovic V, Pico AR, Vailaya A, Wang PL, Adler A, Conklin BR, Hood L, Kuiper M, Sander C, Schmulevich I, Schwikowski B, Warner GJ, Ideker T, Bader GD. Integration of biological networks and gene expression data using Cytoscape. Nat Protoc. 2007; 2:2366-82.

7) Maere S, Heymans K, Kuiper M. BiNGO: a Cytoscape plugin to assess overrepresentation of gene ontology categories in biological networks. Bioinformatics 2005; 21:3448-9.

8) Dennis G Jr, Sherman BT, Hosack DA, Yang J, Gao W, Lane HC, Lempicki RA. DAVID: Database for Annotation, Visualization, and Integrated Discovery. Genome Biol. 2003;4:P3.

9) Durinck S, Spellman P, Birney E and Huber W. Mapping identifiers for the integration of genomic datasets with the R/Bioconductor package biomaRt. Nature Protocols 2009; 4: 1184-1191.

10) Durinck S, Moreau Y, Kasprzyk A, Davis S, De Moor B, Brazma A and Huber W, BioMart and Bioconductor: a powerful link between biological databases and microarray data analysis. Bioinformatics 2005; 21: 3439-3440.

11) Kent WJ, Sugnet CW, Furey TS, Roskin KM, Pringle TH, Zahler AM, Haussler D. The human genome browser at UCSC. Genome Res. 2002;12:996-1006.

12) Fujita PA, Rhead B, Zweig AS, Hinrichs AS, Karolchik D, Cline MS, Goldman M, Barber GP, Clawson H, Coelho A, Diekhans M, Dreszer TR, Giardine BM, Harte RA, Hillman-Jackson J, Hsu F, Kirkup V, Kuhn RM, Learned K, Li CH, Meyer LR, Pohl A, Raney BJ, Rosenbloom KR, Smith KE, Haussler D, Kent WJ. The UCSC Genome Browser database: update 2011.  Nucleic Acids Res. 2012;40 (Database issue):D918-23.

13) ENCODE Project Consortium, Myers RM, Stamatoyannopoulos J, Snyder M, Dunham I, Hardison RC, Bernstein BE, Gingeras TR, Kent WJ, Birney E et al. A user's guide to the encyclopedia of DNA elements (ENCODE). PLoS Biol*.* 2011; 9:e1001046.

14) Smit, AFA, Hubley, R & Green, P.  *RepeatMasker Open-3.0*. 1996-2010 (http://www.repeatmasker.org).

15) Luedi PP, Dietrich FS, Weidman JR, Bosko JM, Jirtle RL, Hartemink AJ. Computational and experimental identification of novel human imprinted genes. Genome Res. 2007; 17:1723-30

16) Fang F, Hodges E, Molaro A, Dean M, Hannon GJ, Smith AD. Genomic landscape of human allele-specific DNA methylation. Proc Natl Acad Sci U S A. 2012; 109:7332-7.

17) Yuen RK, Jiang R, Peñaherrera MS, McFadden DE, Robinson WP. Genome-wide mapping of imprinted differentially methylated regions by DNA methylation profiling of human placentas from triploidies. Epigenetics Chromatin. 2011; 4:10.

18) Court F, Tayama C, Romanelli V, Martin-Trujillo A, Iglesias-Platas I, Okamura K, *et al*. Genome-wide parent-of-origin DNA methylation analysis reveals the intricacies of human imprinting and suggests a germline methylation-independent mechanism of establishment. Genome Res. 2014; 24:554-69.

19) Barbaux S, Gascoin-Lachambre G, Buffat C, Monnier P, Mondon F, Tonanny MB, *et al*. A genome-wide approach reveals novel imprinted genes expressed in the human placenta Epigenetics. 2012; 7:1079-90.

20) Daelemans C, Ritchie ME, Smits G, Abu-Amero S, Sudbery IM, Forrest MS, *et al*. High-throughput analysis of candidate imprinted genes and allele-specific gene expression in the human term placenta. BMC Genet. 2010; 11:25.

21) Das R, Lee YK, Strogantsev R, Jin S, Lim YC, Ng PY, *et al*. DNMT1 and AIM1 Imprinting in human placenta revealed through a genome-wide screen for allele-specific DNA methylation. BMC Genomics. 2013; 14:685.

22) Smith ZD, Chan MM, Humm KC, Karnik R, Mekhoubad S, Regev A, et al.

DNA methylation dynamics of the human preimplantation embryo. Nature 2014; 511:611-5
