# Supplementary material for: Deoxyribonucleic acid methylation profiling of single human blastocysts by methylated CpG-island amplification coupled with CpG-island microarray
Source: Fertil Steril. 2015 Jun;103(6):1566–1571.e4. doi: 10.1016/j.fertnstert.2015.03.020 (PMC4449363; doi:10.1016/j.fertnstert.2015.03.020)
Supplement: Supplemental Table 1 [file mmc2.docx]

**Supplemental Table 1**

**Statistical analysis for the CpG Islands (CGIs) methylated in human blastocysts.**

| **CGIs** | **Observed** | **Expected** | | **Probability based on a binomial model** | | **Chi-Square test *P* value** | |
| --- | --- | --- | --- | --- | --- | --- | --- |
|  |  | **Based on 1,263 CGIs** | **Based on 27,800 CGIs** | **Based on 1,263 CGIs** | **based on 27,800 CGIs** | **Based on 1,263 CGIs** | **Based on 27,800 CGIs** |
| Number methylated in 5/5 samples | 121 | 24 | 0.0001 | 0.02 | 3.7E-09 | <2.2E-16 | <2.2E-16 |
| Number methylated in 4/5 samples | 159 | 150 | 0.02 | 0.12 | 9.03E-07 | 0.64 | <2.2E-16 |

Statistical validation of methylated sites compared to a random null hypothesis: 1,263 was the number of unique sites methylated in at least one blastocyst. Statistics based on this number, rather than the number provided by the 27,800 CGIs on the array, provides a more conservative estimate of significance.
