# Supplementary material for: Deoxyribonucleic acid methylation profiling of single human blastocysts by methylated CpG-island amplification coupled with CpG-island microarray
Source: Fertil Steril. 2015 Jun;103(6):1566–1571.e4. doi: 10.1016/j.fertnstert.2015.03.020 (PMC4449363; doi:10.1016/j.fertnstert.2015.03.020)
Supplement: Supplemental Table 2 [file mmc3.docx]

**Supplemental Table 2**

**Positions of 121 CpG islands that were methylated in all five tested embryos.**

| **Chromosome** | **CGI start position** | **CGI end position** | **Gene symbol** | | **Gene name** | **Methylation of this region in other cell types** |
| --- | --- | --- | --- | --- | --- | --- |
| chr1 | 979447 | 980334 | AGRN | | agrin [Source:HGNC Symbol;Acc:329] | confirmed |
| chr1 | 1019750 | 1020109 | C1orf159 | | chromosome 1 open reading frame 159 [Source:HGNC Symbol;Acc:26062] | confirmed |
| chr1 | 1911939 | 1912557 | C1orf222 | | chromosome 1 open reading frame 222 [Source:HGNC Symbol;Acc:27917] | not confirmed |
| chr1 | 3341973 | 3342487 | PRDM16 | | PR domain containing 16 [Source:HGNC Symbol;Acc:14000] | confirmed |
| chr1 | 3459790 | 3460238 | MEGF6 | | multiple EGF-like-domains 6 [Source:HGNC Symbol;Acc:3232] | not confirmed |
| chr1 | 17866502 | 17866716 | ARHGEF10L | | Rho guanine nucleotide exchange factor (GEF) 10-like [Source:HGNC Symbol;Acc:25540] | not confirmed |
| chr1 | 29563659 | 29563941 | PTPRU | | protein tyrosine phosphatase, receptor type, U [Source:HGNC Symbol;Acc:9683] | not confirmed |
| chr2 | 3063285 | 3063724 |  | |  | confirmed |
| chr2 | 220404622 | 220404959 | DNPEP | | aspartyl aminopeptidase [Source:HGNC Symbol;Acc:2981] | not confirmed |
| chr2 | 240033078 | 240033380 | HDAC4 | | histone deacetylase 4 [Source:HGNC Symbol;Acc:14063] | confirmed |
| chr2 | 240878124 | 240878596 | NDUFA10 | | NADH dehydrogenase (ubiquinone) 1 alpha subcomplex, 10, 42kDa [Source:HGNC Symbol;Acc:7684] | confirmed |
| chr2 | 242148733 | 242149107 | ANO7 | | anoctamin 7 [Source:HGNC Symbol;Acc:31677] | confirmed |
| chr3 | 12976963 | 12977653 | IQSEC1 | | IQ motif and Sec7 domain 1 [Source:HGNC Symbol;Acc:29112] | confirmed |
| chr4 | 1218858 | 1219773 | CTBP1 | | C-terminal binding protein 1 [Source:HGNC Symbol;Acc:2494] | confirmed |
| chr4 | 3516448 | 3516868 | LRPAP1 | | low density lipoprotein receptor-related protein associated protein 1 [Source:HGNC Symbol;Acc:6701] | confirmed |
| chr4 | 154216589 | 154217029 | TRIM2 | | tripartite motif containing 2 [Source:HGNC Symbol;Acc:15974] | confirmed |
| chr5 | 1277419 | 1277680 | TERT | | telomerase reverse transcriptase [Source:HGNC Symbol;Acc:11730] | not confirmed |
| chr5 | 134526887 | 134527281 |  |  | | not confirmed |
| chr5 | 139040819 | 139041040 | CXXC5 | | CXXC finger protein 5 [Source:HGNC Symbol;Acc:26943] | not confirmed |
| chr6 | 1624752 | 1625362 | GMDS | | GDP-mannose 4,6-dehydratase [Source:HGNC Symbol;Acc:4369] | not confirmed |
| chr6 | 160554666 | 160555282 | SLC22A1 | | solute carrier family 22 (organic cation transporter), member 1 [Source:HGNC Symbol;Acc:10963] | confirmed |
| chr6 | 164393116 | 164393583 |  | |  | confirmed |
| chr6 | 169631687 | 169632871 | THBS2 | | thrombospondin 2 [Source:HGNC Symbol;Acc:11786] | confirmed |
| chr7 | 1149740 | 1149989 | C7orf50 | | chromosome 7 open reading frame 50 [Source:HGNC Symbol;Acc:22421] | confirmed |
| chr7 | 1514136 | 1514598 | INTS1 | | integrator complex subunit 1 [Source:HGNC Symbol;Acc:24555] | confirmed |
| chr7 | 1575654 | 1576026 | MAFK | | v-maf musculoaponeurotic fibrosarcoma oncogene homolog K (avian) [Source:HGNC Symbol;Acc:6782] | not confirmed |
| chr7 | 1641710 | 1641956 |  |  | | not confirmed |
| chr7 | 2059866 | 2060170 | MAD1L1 | | MAD1 mitotic arrest deficient-like 1 (yeast) [Source:HGNC Symbol;Acc:6762] | confirmed |
| chr7 | 4801772 | 4802304 | FOXK1 | | forkhead box K1 [Source:HGNC Symbol;Acc:23480] | confirmed |
| chr7 | 57247616 | 57247860 |  |  | | not confirmed |
| chr7 | 66368762 | 66369178 |  | |  | confirmed |
| chr7 | 157577644 | 157577965 | PTPRN2 | | protein tyrosine phosphatase, receptor type, N polypeptide 2 [Source:HGNC Symbol;Acc:9677] | confirmed |
| chr8 | 141108261 | 141110387 | TRAPPC9 | | trafficking protein particle complex 9 [Source:HGNC Symbol;Acc:30832] | confirmed |
| chr8 | 144552498 | 144552796 | ZC3H3 | | zinc finger CCCH-type containing 3 [Source:HGNC Symbol;Acc:28972] | confirmed |
| chr8 | 144994505 | 144995315 | PLEC | | plectin isoform 1e [Source:RefSeq peptide;Acc:NP_958781] | confirmed |
| chr8 | 145830775 | 145831247 | ARHGAP39 | | Rho GTPase activating protein 39 [Source:HGNC Symbol;Acc:29351] | confirmed |
| chr9 | 5041545 | 5041933 | JAK2 | | Janus kinase 2 [Source:HGNC Symbol;Acc:6192] | confirmed |
| chr9 | 66455746 | 66456068 |  |  | | not confirmed |
| chr9 | 96009829 | 96010086 | WNK2 | | WNK lysine deficient protein kinase 2 [Source:HGNC Symbol;Acc:14542] | confirmed |
| chr9 | 138966611 | 138967111 | NACC2 | | NACC family member 2, BEN and BTB (POZ) domain containing [Source:HGNC Symbol;Acc:23846] | confirmed |
| chr9 | 140310808 | 140312032 | EXD3 | | exonuclease 3'-5' domain containing 3 [Source:HGNC Symbol;Acc:26023] | confirmed |
| chr9 | 140395167 | 140395833 | PNPLA7 | | patatin-like phospholipase domain containing 7 [Source:HGNC Symbol;Acc:24768] | confirmed |
| chr10 | 413666 | 413816 | DIP2C | | DIP2 disco-interacting protein 2 homolog C (Drosophila) [Source:HGNC Symbol;Acc:29150] | confirmed |
| chr10 | 105362174 | 105362906 | SH3PXD2A | | SH3 and PX domains 2A [Source:HGNC Symbol;Acc:23664] | confirmed |
| chr10 | 134733478 | 134733849 | TTC40/C6orf93 | | tetratricopeptide repeat domain 40 [Source:HGNC Symbol;Acc:25247] | not confirmed |
| chr11 | 858551 | 858734 | TSPAN4 | | tetraspanin 4 [Source:HGNC Symbol;Acc:11859] | not confirmed |
| chr11 | 1095996 | 1096554 | MUC2 | | mucin 2, oligomeric mucus/gel-forming [Source:HGNC Symbol;Acc:7512] | confirmed |
| chr11 | 64596950 | 64597242 | CDC42BPG | | CDC42 binding protein kinase gamma (DMPK-like) [Source:HGNC Symbol;Acc:29829] | confirmed |
| chr11 | 130343270 | 130343712 | ADAMTS15 | | ADAM metallopeptidase with thrombospondin type 1 motif, 15 [Source:HGNC Symbol;Acc:16305] | not confirmed |
| chr12 | 6857926 | 6858301 | MLF2 | | myeloid leukemia factor 2 [Source:HGNC Symbol;Acc:7126] | confirmed |
| chr12 | 65218615 | 65219169 | TBC1D30 | | TBC1 domain family, member 30 [Source:HGNC Symbol;Acc:29164] | not confirmed |
| chr12 | 120031525 | 120031973 |  | | | not confirmed |
| chr13 | 48892830 | 48893870 | RB1 | | retinoblastoma 1 [Source:HGNC Symbol;Acc:9884] | confirmed |
| chr13 | 113649000 | 113649645 | MCF2L | | MCF.2 cell line derived transforming sequence-like [Source:HGNC Symbol;Acc:14576] | confirmed |
| chr13 | 114523409 | 114524184 | GAS6 | | growth arrest-specific 6 [Source:HGNC Symbol;Acc:4168] | not confirmed |
| chr13 | 114535197 | 114535429 | GAS6 | | growth arrest-specific 6 [Source:HGNC Symbol;Acc:4168] | not confirmed |
| chr14 | 104639189 | 104639944 | KIF26A | | kinesin family member 26A [Source:HGNC Symbol;Acc:20226] | confirmed |
| chr14 | 104643150 | 104644287 | KIF26A | | kinesin family member 26A [Source:HGNC Symbol;Acc:20226] | confirmed |
| chr14 | 105180957 | 105181479 | INF2 | | inverted formin, FH2 and WH2 domain containing [Source:HGNC Symbol;Acc:23791] | confirmed |
| chr14 | 105251319 | 105251641 | AKT1 | | v-akt murine thymoma viral oncogene homolog 1 [Source:HGNC Symbol;Acc:391] | confirmed |
| chr14 | 105684875 | 105685794 | BRF1 | | BRF1 homolog, subunit of RNA polymerase III transcription initiation factor IIIB (S. cerevisiae) [Source:HGNC Symbol;Acc:11551] | confirmed |
| chr14 | 105836006 | 105836385 | PACS2 | | phosphofurin acidic cluster sorting protein 2 [Source:HGNC Symbol;Acc:23794] | confirmed |
| chr14 | 105911712 | 105912156 | MTA1 | | metastasis associated 1 [Source:HGNC Symbol;Acc:7410] | confirmed |
| chr16 | 798173 | 798529 | MSLN | | mesothelin [Source:HGNC Symbol;Acc:7371] | not confirmed |
| chr16 | 1207101 | 1208046 | CACNA1H | | calcium channel, voltage-dependent, T type, alpha 1H subunit [Source:HGNC Symbol;Acc:1395] | confirmed |
| chr16 | 1227200 | 1227545 | CACNA1H | | calcium channel, voltage-dependent, T type, alpha 1H subunit [Source:HGNC Symbol;Acc:1395] | confirmed |
| chr16 | 1583754 | 1584724 | IFT140 | | intraflagellar transport 140 homolog (Chlamydomonas) [Source:HGNC Symbol;Acc:29077] | confirmed |
| chr16 | 1705824 | 1706214 | CRAMP1L | | Crm, cramped-like (Drosophila) [Source:HGNC Symbol;Acc:14122] | confirmed |
| chr16 | 1836656 | 1837038 |  | |  | confirmed |
| chr16 | 1840622 | 1842174 | IGFALS | | insulin-like growth factor binding protein, acid labile subunit [Source:HGNC Symbol;Acc:5468] | confirmed |
| chr16 | 29997592 | 29997939 | TAOK2 | | TAO kinase 2 [Source:HGNC Symbol;Acc:16835] | confirmed |
| chr16 | 29998759 | 29999036 | TAOK2 | | TAO kinase 2 [Source:HGNC Symbol;Acc:16835] | confirmed |
| chr16 | 33936405 | 33936748 |  | |  | confirmed |
| chr16 | 75268992 | 75269772 | BCAR1 | | breast cancer anti-estrogen resistance 1 [Source:HGNC Symbol;Acc:971] | confirmed |
| chr16 | 85529750 | 85530215 |  | |  | confirmed |
| chr16 | 87741876 | 87742187 | KLHDC4 | | kelch domain containing 4 [Source:HGNC Symbol;Acc:25272] | confirmed |
| chr16 | 88165537 | 88166121 |  | |  | confirmed |
| chr16 | 88951350 | 88951701 | CBFA2T3 | | core-binding factor, runt domain, alpha subunit 2; translocated to, 3 [Source:HGNC Symbol;Acc:1537] | confirmed |
| chr16 | 89186216 | 89186598 | ACSF3 | | acyl-CoA synthetase family member 3 [Source:HGNC Symbol;Acc:27288] | confirmed |
| chr17 | 5973237 | 5973520 |  |  | | not confirmed |
| chr17 | 21318598 | 21319403 |  |  | | not confirmed |
| chr17 | 65040710 | 65040955 | CACNG1 | | calcium channel, voltage-dependent, gamma subunit 1 [Source:HGNC Symbol;Acc:1405] | confirmed |
| chr17 | 79374042 | 79374765 | BAHCC1 | | BAH domain and coiled-coil containing 1 [Source:HGNC Symbol;Acc:29279] | not confirmed |
| chr17 | 79944572 | 79944866 | ASPSCR1 | | alveolar soft part sarcoma chromosome region, candidate 1 [Source:HGNC Symbol;Acc:13825] | confirmed |
| chr17 | 79945242 | 79945358 | ASPSCR1 | | alveolar soft part sarcoma chromosome region, candidate 1 [Source:HGNC Symbol;Acc:13825] | confirmed |
| chr17 | 79967025 | 79967192 | ASPSCR1 | | alveolar soft part sarcoma chromosome region, candidate 1 [Source:HGNC Symbol;Acc:13825] | confirmed |
| chr17 | 80887896 | 80888317 | TBCD | | tubulin folding cofactor D [Source:HGNC Symbol;Acc:11581] | confirmed |
| chr17 | 80918369 | 80919324 | B3GNTL1 | | UDP-GlcNAc:betaGal beta-1,3-N-acetylglucosaminyltransferase-like 1 [Source:HGNC Symbol;Acc:21727] | confirmed |
| chr18 | 2913003 | 2913361 | EMILIN2 | | elastin microfibril interfacer 2 [Source:HGNC Symbol;Acc:19881] | confirmed |
| chr18 | 77309626 | 77309926 |  |  | | not confirmed |
| chr18 | 77312658 | 77313033 |  |  | | not confirmed |
| chr18 | 77495497 | 77496354 | CTDP1 | | CTD (carboxy-terminal domain, RNA polymerase II, polypeptide A) phosphatase, subunit 1 [Source:HGNC Symbol;Acc:2498] | confirmed |
| chr18 | 77507531 | 77507861 | CTDP1 | | CTD (carboxy-terminal domain, RNA polymerase II, polypeptide A) phosphatase, subunit 1 [Source:HGNC Symbol;Acc:2498] | confirmed |
| chr19 | 418949 | 419207 | SHC2 | | SHC (Src homology 2 domain containing) transforming protein 2 [Source:HGNC Symbol;Acc:29869] | confirmed |
| chr19 | 539014 | 539556 | CDC34 | | cell division cycle 34 homolog (S. cerevisiae) [Source:HGNC Symbol;Acc:1734] | confirmed |
| chr19 | 577762 | 578510 | BSG | | basigin (Ok blood group) [Source:HGNC Symbol;Acc:1116] | confirmed |
| chr19 | 579156 | 579820 | BSG | | basigin (Ok blood group) [Source:HGNC Symbol;Acc:1116] | confirmed |
| chr19 | 644616 | 644779 |  | |  | confirmed |
| chr19 | 1118941 | 1120242 | SBNO2 | | strawberry notch homolog 2 (Drosophila) [Source:HGNC Symbol;Acc:29158] | confirmed |
| chr19 | 1395291 | 1395835 | NDUFS7 | | NADH dehydrogenase (ubiquinone) Fe-S protein 7, 20kDa (NADH-coenzyme Q reductase) [Source:HGNC Symbol;Acc:7714] | confirmed |
| chr19 | 1818388 | 1818849 | REXO1 | | REX1, RNA exonuclease 1 homolog (S. cerevisiae) [Source:HGNC Symbol;Acc:24616] | confirmed |
| chr19 | 1947703 | 1948128 | CSNK1G2 | | casein kinase 1, gamma 2 [Source:HGNC Symbol;Acc:2455] | confirmed |
| chr19 | 4032921 | 4033665 | PIAS4 | | protein inhibitor of activated STAT, 4 [Source:HGNC Symbol;Acc:17002] | confirmed |
| chr19 | 11170575 | 11170846 | SMARCA4 | | SWI/SNF related, matrix associated, actin dependent regulator of chromatin, subfamily a, member 4 [Source:HGNC Symbol;Acc:11100] | confirmed |
| chr19 | 19416671 | 19416903 | SUGP1 | | SURP and G patch domain containing 1 [Source:HGNC Symbol;Acc:18643] | confirmed |
| chr20 | 60968936 | 60969320 | CABLES2 | | Cdk5 and Abl enzyme substrate 2 [Source:HGNC Symbol;Acc:16143] | confirmed |
| chr20 | 61512172 | 61513790 | DIDO1 | | death inducer-obliterator 1 [Source:HGNC Symbol;Acc:2680] | confirmed |
| chr20 | 62221306 | 62222078 | GMEB2 | | glucocorticoid modulatory element binding protein 2 [Source:HGNC Symbol;Acc:4371] | confirmed |
| chr20 | 62729648 | 62730218 | OPRL1 | | opiate receptor-like 1 [Source:HGNC Symbol;Acc:8155] | confirmed |
| chr20 | 57465876 | 57466115 | GNAS | | GNAS complex locus [Source:HGNC Symbol;Acc:4392] | not confirmed |
| chr21 | 46850550 | 46850871 | COL18A1 | | collagen, type XVIII, alpha 1 [Source:HGNC Symbol;Acc:2195] | not confirmed |
| chr21 | 47421822 | 47422968 | COL6A1 | | collagen, type VI, alpha 1 [Source:HGNC Symbol;Acc:2211] | confirmed |
| chr22 | 37911912 | 37912256 | CARD10 | | caspase recruitment domain family, member 10 [Source:HGNC Symbol;Acc:16422] | confirmed |
| chr22 | 50720237 | 50721785 | PLXNB2 | | plexin B2 [Source:HGNC Symbol;Acc:9104] | confirmed |
| chr22 | 51142749 | 51143148 | SHANK3 | | SH3 and multiple ankyrin repeat domains 3 [Source:HGNC Symbol;Acc:14294] | confirmed |
| chrX | 227606 | 228327 | GTPBP6 | | GTP binding protein 6 (putative) [Source:HGNC Symbol;Acc:30189] | not confirmed |
| chrX | 323897 | 324541 | PPP2R3B | | protein phosphatase 2, regulatory subunit B'', beta [Source:HGNC Symbol;Acc:13417] | not confirmed |
| chrX | 460941 | 461540 |  |  | | not confirmed |
| chrX | 1716958 | 1718689 | AKAP17A | | A kinase (PRKA) anchor protein 17A [Source:HGNC Symbol;Acc:18783] | not confirmed |
| chrX | 2408471 | 2409085 | DHRSX | | dehydrogenase/reductase (SDR family) X-linked [Source:HGNC Symbol;Acc:18399] | not confirmed |
| chrX | 9863510 | 9863765 | SHROOM2 | | shroom family member 2 [Source:HGNC Symbol;Acc:630] | confirmed |

The far right hand column identifies the methylated CpG islands in blastocysts that corresponded genomic regions that are known to be methylated in other human cell types via the ENCODE project (GM12878, HI-hESC, HeLa-S3, HUVEC, K562, HMEC and HepG2. Those sites that do not correspond are labelled ‘not confirmed’, all other sites were matched (confirmed).
