# Supplementary material for: Deoxyribonucleic acid methylation profiling of single human blastocysts by methylated CpG-island amplification coupled with CpG-island microarray
Source: Fertil Steril. 2015 Jun;103(6):1566–1571.e4. doi: 10.1016/j.fertnstert.2015.03.020 (PMC4449363; doi:10.1016/j.fertnstert.2015.03.020)
Supplement: Supplemental Table 3 [file mmc4.docx]

**Supplemental Table 3**

**Positions of 159 CpG islands that were methylated in four of the five tested embryos.**

| **Chromosome** | **CGI start position** | **CGI end position** | **Gene symbol** | **Gene name** | **Methylation of this region in other cell types** |
| --- | --- | --- | --- | --- | --- |
| chr1 | 1016801 | 1017513 | C1orf159 | chromosome 1 open reading frame 159 [Source:HGNC Symbol;Acc:26062] | confirmed |
| chr1 | 1203146 | 1203661 | UBE2J2 | ubiquitin-conjugating enzyme E2, J2 [Source:HGNC Symbol;Acc:19268] | confirmed |
| chr1 | 1470586 | 1471355 | TMEM240 | transmembrane protein 240 [Source:HGNC Symbol;Acc:25186] | confirmed |
| chr1 | 2316272 | 2316747 | MORN1 | MORN repeat containing 1 [Source:HGNC Symbol;Acc:25852] | confirmed |
| chr1 | 2411084 | 2411426 | PLCH2 | phospholipase C, eta 2 [Source:HGNC Symbol;Acc:29037] | confirmed |
| chr1 | 3102494 | 3103389 | PRDM16 | PR domain containing 16 [Source:HGNC Symbol;Acc:14000] | confirmed |
| chr1 | 7723316 | 7725078 | CAMTA1 | calmodulin binding transcription activator 1 [Source:HGNC Symbol;Acc:18806] | confirmed |
| chr1 | 16270322 | 16271598 | ZBTB17 | zinc finger and BTB domain containing 17 [Source:HGNC Symbol;Acc:12936] | confirmed |
| chr1 | 35334306 | 35334577 | DLGAP3 | discs, large (Drosophila) homolog-associated protein 3 [Source:HGNC Symbol;Acc:30368] | confirmed |
| chr1 | 90309090 | 90309629 | LRRC8C | leucine rich repeat containing 8 family, member C [Source:HGNC Symbol;Acc:25075] | not confirmed |
| chr1 | 108113150 | 108113419 |  |  | confirmed |
| chr1 | 171640045 | 171640268 |  |  | confirmed |
| chr1 | 228503542 | 228504117 | OBSCN | obscurin, cytoskeletal calmodulin and titin-interacting RhoGEF [Source:HGNC Symbol;Acc:15719] | confirmed |
| chr1 | 245848614 | 245849050 | KIF26B | kinesin family member 26B [Source:HGNC Symbol;Acc:25484] | confirmed |
| chr1 | 247614632 | 247614984 | OR2B11 | olfactory receptor, family 2, subfamily B, member 11 [Source:HGNC Symbol;Acc:31249] | confirmed |
| chr2 | 92260801 | 92261008 |  |  | not confirmed |
| chr2 | 241535642 | 241536016 | CAPN10 | calpain 10 [Source:HGNC Symbol;Acc:1477] | confirmed |
| chr3 | 48677572 | 48677915 | CELSR3 | cadherin, EGF LAG seven-pass G-type receptor 3 (flamingo homolog, Drosophila) [Source:HGNC Symbol;Acc:3230] | confirmed |
| chr3 | 53032836 | 53033139 | SFMBT1 | Scm-like with four mbt domains 1 [Source:HGNC Symbol;Acc:20255] | confirmed |
| chr3 | 58109054 | 58109406 | FLNB | filamin B, beta [Source:HGNC Symbol;Acc:3755] | confirmed |
| chr4 | 650596 | 651005 | PDE6B | phosphodiesterase 6B, cGMP-specific, rod, beta [Source:HGNC Symbol;Acc:8786] | not confirmed |
| chr4 | 1801032 | 1801390 | FGFR3 | fibroblast growth factor receptor 3 [Source:HGNC Symbol;Acc:3690] | confirmed |
| chr4 | 1987592 | 1988327 |  |  | confirmed |
| chr4 | 1994590 | 1994865 | WHSC2 | Wolf-Hirschhorn syndrome candidate 2 [Source:HGNC Symbol;Acc:12768] | confirmed |
| chr4 | 64214820 | 64215589 |  |  | not confirmed |
| chr4 | 187810529 | 187810758 |  |  | confirmed |
| chr5 | 494767 | 495180 | SLC9A3 | solute carrier family 9 (sodium/hydrogen exchanger), member 3 [Source:HGNC Symbol;Acc:11073] | confirmed |
| chr5 | 498080 | 498464 | SLC9A3 | solute carrier family 9 (sodium/hydrogen exchanger), member 3 [Source:HGNC Symbol;Acc:11073] | confirmed |
| chr5 | 1038023 | 1038406 | NKD2 | naked cuticle homolog 2 (Drosophila) [Source:HGNC Symbol;Acc:17046] | confirmed |
| chr5 | 1073537 | 1074104 | SLC12A7 | solute carrier family 12 (potassium/chloride transporters), member 7 [Source:HGNC Symbol;Acc:10915] | confirmed |
| chr5 | 1077859 | 1078100 | SLC12A7 | solute carrier family 12 (potassium/chloride transporters), member 7 [Source:HGNC Symbol;Acc:10915] | confirmed |
| chr5 | 141262925 | 141263266 |  |  | not confirmed |
| chr5 | 178684275 | 178684706 | ADAMTS2 | ADAM metallopeptidase with thrombospondin type 1 motif, 2 [Source:HGNC Symbol;Acc:218] | confirmed |
| chr5 | 179306069 | 179306354 | TBC1D9B | TBC1 domain family, member 9B (with GRAM domain) [Source:HGNC Symbol;Acc:29097] | confirmed |
| chr6 | 3849242 | 3849749 | FAM50B | family with sequence similarity 50, member B [Source:HGNC Symbol;Acc:18789] | not confirmed |
| chr6 | 37626031 | 37626355 | MDGA1 | MAM domain containing glycosylphosphatidylinositol anchor 1 [Source:HGNC Symbol;Acc:19267] | confirmed |
| chr6 | 166825864 | 166826503 | RPS6KA2 | ribosomal protein S6 kinase, 90kDa, polypeptide 2 [Source:HGNC Symbol;Acc:10431] | confirmed |
| chr6 | 170059159 | 170059624 | WDR27 | WD repeat domain 27 [Source:HGNC Symbol;Acc:21248] | confirmed |
| chr7 | 947362 | 947691 | ADAP1 | ArfGAP with dual PH domains 1 [Source:HGNC Symbol;Acc:16486] | confirmed |
| chr7 | 1532970 | 1533221 | INTS1 | integrator complex subunit 1 [Source:HGNC Symbol;Acc:24555] | confirmed |
| chr7 | 1587237 | 1587755 | TMEM184A | transmembrane protein 184A [Source:HGNC Symbol;Acc:28797] | confirmed |
| chr7 | 1908530 | 1908829 | MAD1L1 | MAD1 mitotic arrest deficient-like 1 (yeast) [Source:HGNC Symbol;Acc:6762] | confirmed |
| chr7 | 1913761 | 1914147 | MAD1L1 | MAD1 mitotic arrest deficient-like 1 (yeast) [Source:HGNC Symbol;Acc:6762] | confirmed |
| chr7 | 2632167 | 2632406 | IQCE | IQ motif containing E [Source:HGNC Symbol;Acc:29171] | confirmed |
| chr7 | 2645497 | 2645788 | IQCE | IQ motif containing E [Source:HGNC Symbol;Acc:29171] | confirmed |
| chr7 | 4153646 | 4153929 | SDK1 | sidekick cell adhesion molecule 1 [Source:HGNC Symbol;Acc:19307] | confirmed |
| chr7 | 4800542 | 4800971 | FOXK1 | forkhead box K1 [Source:HGNC Symbol;Acc:23480] | confirmed |
| chr7 | 157352984 | 157353295 | PTPRN2 | protein tyrosine phosphatase, receptor type, N polypeptide 2 [Source:HGNC Symbol;Acc:9677] | confirmed |
| chr7 | 157448251 | 157448654 | PTPRN2 | protein tyrosine phosphatase, receptor type, N polypeptide 2 [Source:HGNC Symbol;Acc:9677] | confirmed |
| chr8 | 1443833 | 1444130 |  |  | confirmed |
| chr8 | 1496686 | 1497066 | DLGAP2 | discs, large (Drosophila) homolog-associated protein 2 [Source:HGNC Symbol;Acc:2906] | confirmed |
| chr8 | 144885216 | 144885484 | SCRIB | protein scribble homolog isoform a [Source:RefSeq peptide;Acc:NP_874365 ]scribbled homolog (Drosophila) [Source:HGNC Symbol;Acc:30377] | not confirmed |
| chr8 | 144995639 | 144997684 |  | PLEC plectin isoform 1e [Source:RefSeq peptide;Acc:NP_958781] | confirmed |
| chr8 | 145024438 | 145024876 |  | PLEC plectin isoform 1e [Source:RefSeq peptide;Acc:NP_958781] | confirmed |
| chr9 | 2241394 | 2241930 |  |  | not confirmed |
| chr9 | 2933404 | 2933780 |  |  | confirmed |
| chr9 | 34989473 | 34989916 | DNAJB5 | DnaJ (Hsp40) homolog, subfamily B, member 5 [Source:HGNC Symbol;Acc:14887] | not confirmed |
| chr9 | 136677243 | 136677605 | VAV2 | vav 2 guanine nucleotide exchange factor [Source:HGNC Symbol;Acc:12658] | confirmed |
| chr9 | 137734094 | 137734536 | COL5A1 | collagen, type V, alpha 1 [Source:HGNC Symbol;Acc:2209] | confirmed |
| chr9 | 138662623 | 138662933 | KCNT1 | potassium channel, subfamily T, member 1 [Source:HGNC Symbol;Acc:18865] | not confirmed |
| chr9 | 139262159 | 139262455 | CARD9 | caspase recruitment domain family, member 9 [Source:HGNC Symbol;Acc:16391] | not confirmed |
| chr9 | 140248219 | 140248639 | EXD3 | exonuclease 3'-5' domain containing 3 [Source:HGNC Symbol;Acc:26023] | not confirmed |
| chr9 | 140349704 | 140349993 | NELF | nasal embryonic LHRH factor [Source:HGNC Symbol;Acc:29843] | confirmed |
| chr9 | 140777137 | 140777484 | CACNA1B | calcium channel, voltage-dependent, N type, alpha 1B subunit [Source:HGNC Symbol;Acc:1389] | not confirmed |
| chr9 | 141014620 | 141014925 | CACNA1B | calcium channel, voltage-dependent, N type, alpha 1B subunit [Source:HGNC Symbol;Acc:1389] | not confirmed |
| chr10 | 71892050 | 71892405 | AIFM2 | apoptosis-inducing factor, mitochondrion-associated, 2 [Source:HGNC Symbol;Acc:21411] | not confirmed |
| chr10 | 106074920 | 106075598 | ITPRIP | inositol 1,4,5-trisphosphate receptor interacting protein [Source:HGNC Symbol;Acc:29370] | confirmed |
| chr10 | 126281382 | 126281638 | LHPP | phospholysine phosphohistidine inorganic pyrophosphate phosphatase [Source:HGNC Symbol;Acc:30042] | confirmed |
| chr10 | 128993467 | 128994033 | DOCK1  /FAM196A | dedicator of cytokinesis 1 [Source:HGNC Symbol;Acc:2987] | not confirmed |
| chr10 | 131592323 | 131592655 |  |  | confirmed |
| chr10 | 134729740 | 134730056 | TTC40/  C6orf93 | tetratricopeptide repeat domain 40 [Source:HGNC Symbol;Acc:25247] | confirmed |
| chr10 | 135082038 | 135082431 | ADAM8 | ADAM metallopeptidase domain 8 [Source:HGNC Symbol;Acc:215] | confirmed |
| chr10 | 135273013 | 135273230 |  |  | confirmed |
| chr11 | 1316361 | 1317064 | TOLLIP | toll interacting protein [Source:HGNC Symbol;Acc:16476] | confirmed |
| chr11 | 1483688 | 1483980 | BRSK2 | BR serine/threonine kinase 2 [Source:HGNC Symbol;Acc:11405] | confirmed |
| chr11 | 8752313 | 8752596 | ST5 | suppression of tumorigenicity 5 [Source:HGNC Symbol;Acc:11350] | confirmed |
| chr11 | 66468329 | 66468629 | SPTBN2 | spectrin, beta, non-erythrocytic 2 [Source:HGNC Symbol;Acc:11276] | confirmed |
| chr11 | 68190952 | 68191243 | LRP5 | low density lipoprotein receptor-related protein 5 [Source:HGNC Symbol;Acc:6697] | confirmed |
| chr11 | 68192426 | 68192742 | LRP5 | low density lipoprotein receptor-related protein 5 [Source:HGNC Symbol;Acc:6697] | confirmed |
| chr12 | 4479776 | 4479964 | FGF23 | fibroblast growth factor 23 [Source:HGNC Symbol;Acc:3680] | confirmed |
| chr12 | 120032259 | 120033223 |  |  | not confirmed |
| chr12 | 133010598 | 133010999 |  |  | not confirmed |
| chr12 | 133125634 | 133126042 | FBRSL1 | fibrosin-like 1 [Source:HGNC Symbol;Acc:29308] | confirmed |
| chr13 | 113771822 | 113772122 | F7 | coagulation factor VII (serum prothrombin conversion accelerator) [Source:HGNC Symbol;Acc:3544] | confirmed |
| chr13 | 114507521 | 114508018 | FAM70B | family with sequence similarity 70, member B [Source:HGNC Symbol;Acc:28297] | not confirmed |
| chr13 | 114538364 | 114538732 | GAS6 | growth arrest-specific 6 [Source:HGNC Symbol;Acc:4168] | not confirmed |
| chr14 | 100118792 | 100119196 | HHIPL1 | HHIP-like 1 [Source:HGNC Symbol;Acc:19710] | confirmed |
| chr14 | 103550417 | 103550797 |  |  | not confirmed |
| chr14 | 105355772 | 105356098 | KIAA0284 | KIAA0284 [Source:HGNC Symbol;Acc:20362] | confirmed |
| chr15 | 28148222 | 28148536 | OCA2 | oculocutaneous albinism II [Source:HGNC Symbol;Acc:8101] | not confirmed |
| chr15 | 75500774 | 75501225 | C15orf39 | chromosome 15 open reading frame 39 [Source:HGNC Symbol;Acc:24497] | confirmed |
| chr16 | 354244 | 354502 | AXIN1 | axin 1 [Source:HGNC Symbol;Acc:903] | confirmed |
| chr16 | 595795 | 596282 | SOLH | small optic lobes homolog (Drosophila) [Source:HGNC Symbol;Acc:11182] | confirmed |
| chr16 | 841315 | 841920 | CHTF18 | CTF18, chromosome transmission fidelity factor 18 homolog (S. cerevisiae) [Source:HGNC Symbol;Acc:18435] | confirmed |
| chr16 | 1110911 | 1111262 |  |  | confirmed |
| chr16 | 1221786 | 1222296 | CACNA1H | calcium channel, voltage-dependent, T type, alpha 1H subunit [Source:HGNC Symbol;Acc:1395] | confirmed |
| chr16 | 1363739 | 1364500 | UBE2I | ubiquitin-conjugating enzyme E2I [Source:HGNC Symbol;Acc:12485] | confirmed |
| chr16 | 1510834 | 1511474 | CLCN7 | chloride channel 7 [Source:HGNC Symbol;Acc:2025] | confirmed |
| chr16 | 1555020 | 1555277 | TELO2 | TEL2, telomere maintenance 2, homolog (S. cerevisiae) [Source:HGNC Symbol;Acc:29099] | confirmed |
| chr16 | 2757505 | 2757976 | KCTD5 | potassium channel tetramerisation domain containing 5 [Source:HGNC Symbol;Acc:21423] | confirmed |
| chr16 | 70506990 | 70507268 | FUK | fucokinase [Source:HGNC Symbol;Acc:29500] | confirmed |
| chr16 | 75512524 | 75513746 | TMEM170A | transmembrane protein 170A [Source:HGNC Symbol;Acc:29577] | confirmed |
| chr16 | 85674123 | 85674891 | KIAA0182 | KIAA0182 [Source:HGNC Symbol;Acc:28979] | confirmed |
| chr16 | 86565288 | 86565853 | MTHFSD | methenyltetrahydrofolate synthetase domain containing [Source:HGNC Symbol;Acc:25778] | confirmed |
| chr16 | 88815906 | 88816062 | PIEZO1 | piezo-type mechanosensitive ion channel component 1 [Source:HGNC Symbol;Acc:28993] | confirmed |
| chr16 | 88994087 | 88994481 | CBFA2T3 | core-binding factor, runt domain, alpha subunit 2; translocated to, 3 [Source:HGNC Symbol;Acc:1537] | confirmed |
| chr16 | 89977561 | 89977928 | TCF25 | transcription factor 25 (basic helix-loop-helix) [Source:HGNC Symbol;Acc:29181] | confirmed |
| chr17 | 185040 | 185368 | RPH3AL | rabphilin 3A-like (without C2 domains) [Source:HGNC Symbol;Acc:10296] | confirmed |
| chr17 | 1538412 | 1538903 | SCARF1 | scavenger receptor class F, member 1 [Source:HGNC Symbol;Acc:16820] | confirmed |
| chr17 | 6797404 | 6797722 |  |  | not confirmed |
| chr17 | 78081310 | 78081681 | GAA | glucosidase, alpha; acid [Source:HGNC Symbol;Acc:4065] | confirmed |
| chr17 | 79958041 | 79958369 | ASPSCR1 | alveolar soft part sarcoma chromosome region, candidate 1 [Source:HGNC Symbol;Acc:13825] | confirmed |
| chr17 | 80041007 | 80042110 | FASN | fatty acid synthase [Source:HGNC Symbol;Acc:3594] | confirmed |
| chr18 | 55020100 | 55020593 | ST8SIA3 | ST8 alpha-N-acetyl-neuraminide alpha-2,8-sialyltransferase 3 [Source:HGNC Symbol;Acc:14269] | not confirmed |
| chr18 | 77246329 | 77247901 | NFATC1 | nuclear factor of activated T-cells, cytoplasmic, calcineurin-dependent 1 [Source:HGNC Symbol;Acc:7775] | confirmed |
| chr18 | 77308051 | 77308819 |  |  | confirmed |
| chr19 | 536206 | 536842 | CDC34 | cell division cycle 34 homolog (S. cerevisiae) [Source:HGNC Symbol;Acc:1734] | confirmed |
| chr19 | 1110333 | 1110698 | SBNO2 | strawberry notch homolog 2 (Drosophila) [Source:HGNC Symbol;Acc:29158] | confirmed |
| chr19 | 1584477 | 1584904 | MBD3 | methyl-CpG binding domain protein 3 [Source:HGNC Symbol;Acc:6918] | confirmed |
| chr19 | 1625293 | 1625595 | TCF3 | transcription factor 3 (E2A immunoglobulin enhancer binding factors E12/E47) [Source:HGNC Symbol;Acc:11633] | confirmed |
| chr19 | 1828091 | 1828630 | REXO1 | REX1, RNA exonuclease 1 homolog (S. cerevisiae) [Source:HGNC Symbol;Acc:24616] | confirmed |
| chr19 | 2115173 | 2115805 | AP3D1 | adaptor-related protein complex 3, delta 1 subunit [Source:HGNC Symbol;Acc:568] | confirmed |
| chr19 | 3112593 | 3113186 | GNA11 | guanine nucleotide binding protein (G protein), alpha 11 (Gq class) [Source:HGNC Symbol;Acc:4379] | confirmed |
| chr19 | 3198766 | 3199143 | NCLN | nicalin [Source:HGNC Symbol;Acc:26923] | confirmed |
| chr19 | 3648644 | 3649041 | PIP5K1C | phosphatidylinositol-4-phosphate 5-kinase, type I, gamma [Source:HGNC Symbol;Acc:8996] | confirmed |
| chr19 | 3825076 | 3825554 | ZFR2 | zinc finger RNA binding protein 2 [Source:HGNC Symbol;Acc:29189] | confirmed |
| chr19 | 4100979 | 4102471 | MAP2K2 | mitogen-activated protein kinase kinase 2 [Source:HGNC Symbol;Acc:6842] | confirmed |
| chr19 | 5994755 | 5995090 | RFX2 | regulatory factor X, 2 (influences HLA class II expression) [Source:HGNC Symbol;Acc:9983] | confirmed |
| chr19 | 6212318 | 6212931 | MLLT1 | myeloid/lymphoid or mixed-lineage leukemia (trithorax homolog, Drosophila); translocated to, 1 [Source:HGNC Symbol;Acc:7134] | confirmed |
| chr19 | 10304923 | 10305280 | DNMT1 | DNA (cytosine-5-)-methyltransferase 1 [Source:HGNC Symbol;Acc:2976] | not confirmed |
| chr19 | 11098350 | 11098681 | SMARCA4 | SWI/SNF related, matrix associated, actin dependent regulator of chromatin, subfamily a, member 4 [Source:HGNC Symbol;Acc:11100] | confirmed |
| chr19 | 38573401 | 38573733 | SIPA1L3 | signal-induced proliferation-associated 1 like 3 [Source:HGNC Symbol;Acc:23801] | not confirmed |
| chr19 | 46526024 | 46526359 | PGLYRP1 | peptidoglycan recognition protein 1 [Source:HGNC Symbol;Acc:8904] | confirmed |
| chr19 | 54058141 | 54058313 | ZNF331 | zinc finger protein 331 [Source:HGNC Symbol;Acc:15489] | not confirmed |
| chr20 | 57426722 | 57427107 | GNAS | GNAS complex locus [Source:HGNC Symbol;Acc:4392] | not confirmed |
| chr20 | 57797157 | 57797443 | ZNF831 | zinc finger protein 831 [Source:HGNC Symbol;Acc:16167] | not confirmed |
| chr20 | 60926761 | 60927133 | LAMA5 | laminin, alpha 5 [Source:HGNC Symbol;Acc:6485] | confirmed |
| chr20 | 61980486 | 61981024 | CHRNA4 | cholinergic receptor, nicotinic, alpha 4 [Source:HGNC Symbol;Acc:1958] | not confirmed |
| chr20 | 62051918 | 62052327 | KCNQ2 | potassium voltage-gated channel, KQT-like subfamily, member 2 [Source:HGNC Symbol;Acc:6296] | confirmed |
| chr20 | 62193954 | 62194621 | NA | Peroxisomal proliferator-activated receptor A-interacting complex 285 kDa protein [Source:UniProtKB/Swiss-Prot;Acc:Q9BYK8] | not confirmed |
| chr20 | 62195313 | 62197818 | PRIC285 | Peroxisomal proliferator-activated receptor A-interacting complex 285 kDa protein [Source:UniProtKB/Swiss-Prot;Acc:Q9BYK8] | confirmed |
| chr20 | 62493437 | 62493855 | ABHD16B | abhydrolase domain containing 16B [Source:HGNC Symbol;Acc:16128] | not confirmed |
| chr21 | 44088764 | 44089073 | PDE9A | phosphodiesterase 9A [Source:HGNC Symbol;Acc:8795] | confirmed |
| chr21 | 44840105 | 44840533 | SIK1 | salt-inducible kinase 1 [Source:HGNC Symbol;Acc:11142] | confirmed |
| chr21 | 45363489 | 45363887 | AGPAT3 | 1-acylglycerol-3-phosphate O-acyltransferase 3 [Source:HGNC Symbol;Acc:326] | confirmed |
| chr21 | 45742491 | 45742884 | PFKL | phosphofructokinase, liver [Source:HGNC Symbol;Acc:8876] | confirmed |
| chr21 | 47808629 | 47809120 | PCNT | pericentrin [Source:HGNC Symbol;Acc:16068] | confirmed |
| chr22 | 31301620 | 31302204 | MTMR3 | myotubularin related protein 3 [Source:HGNC Symbol;Acc:7451] | confirmed |
| chr22 | 31500993 | 31501270 |  |  | not confirmed |
| chr22 | 40075015 | 40075302 | CACNA1I | calcium channel, voltage-dependent, T type, alpha 1I subunit [Source:HGNC Symbol;Acc:1396] | confirmed |
| chr22 | 45312182 | 45312511 | PHF21B | PHD finger protein 21B [Source:HGNC Symbol;Acc:25161] | not confirmed |
| chr22 | 49145738 | 49147189 | FAM19A5 | family with sequence similarity 19 (chemokine (C-C motif)-like), member A5 [Source:HGNC Symbol;Acc:21592] | confirmed |
| chr22 | 50725380 | 50726601 | PLXNB2 | plexin B2 [Source:HGNC Symbol;Acc:9104]chr1 | confirmed |
| chrX | 192325 | 193340 | PLCXD1 | phosphatidylinositol-specific phospholipase C, X domain containing 1 [Source:HGNC Symbol;Acc:23148] | not confirmed |
| chrX | 305579 | 305837 | PPP2R3B | protein phosphatase 2, regulatory subunit B'', beta [Source:HGNC Symbol;Acc:13417] | not confirmed |
| chrX | 307522 | 308151 | PPP2R3B | protein phosphatase 2, regulatory subunit B'', beta [Source:HGNC Symbol;Acc:13417] | not confirmed |
| chrX | 379831 | 380401 |  |  | not confirmed |
| chrX | 110867405 | 110867671 |  |  | confirmed |
| chrX | 153585657 | 153585999 | FLNA | filamin A, alpha [Source:HGNC Symbol;Acc:3754] | confirmed |

The far right hand column identifies the methylated CpG islands in blastocysts that corresponded genomic regions that are known to be methylated in other human cell types via the ENCODE project (GM12878, HI-hESC, HeLa-S3, HUVEC, K562, HMEC and HepG2. Those sites that do not correspond are labelled ‘not confirmed’; all other sites were matched (confirmed).
