# Supplementary material for: Deoxyribonucleic acid methylation profiling of single human blastocysts by methylated CpG-island amplification coupled with CpG-island microarray
Source: Fertil Steril. 2015 Jun;103(6):1566–1571.e4. doi: 10.1016/j.fertnstert.2015.03.020 (PMC4449363; doi:10.1016/j.fertnstert.2015.03.020)
Supplement: Supplemental Table 4 [file mmc5.docx]

**Supplemental Table 4**

**Gene ontology report for the CGIs that are methylated in all 5 blastocysts that were confirmed as regions of genomic methylation in human cell lines as detailed by the ENCODE project consortium.**

| **GO-ID** | **Description** | **I value** | **Corrected *P* value** | **Genes** |
| --- | --- | --- | --- | --- |
| 5515 | Protein binding | 6.86E-06 | 9.85E-03 | MAD1L1\|BCAR1\|CBFA2T3\|AKT1\|NDUFS7\|TRIM2\|DIP2C\|CABLES2\|SHC2\|SLC22A1\|CTBP1\|BSG\|SH3PXD2A\|BRF1\|CSNK1G2\|PLXNB2\|PTPRN2\|MTA1\|IGFALS\|EXD3\|RB1\|WNK2\|KLHDC4\|CARD10\|LRPAP1\|PIAS4\|TBCD\|KIF26A\|CTDP1\|SMARCA4\|SHROOM2\|CDC34\|PRDM16\|DIDO1\|ARHGAP39\|MLF2\|COL6A1\|AGRN\|THBS2\|PLEC\|INF2\|ASPSCR1\|C7ORF50\|MUC2\|NACC2\|OPRL1\|EMILIN2\|SHANK3\|HDAC4\|CDC42BPG\|JAK2\ |
| 17053 | Transcriptional repressor complex | 1.55E-05 | 1.11E-02 | HDAC4\|CTBP1\|MTA1\|PRDM16 |
| 43233 | Organelle lumen | 7.68E-05 | 3.63E-02 | PACS2\|CTBP1\|NACC2\|BRF1\|FOXK1\|INTS1\|MTA1\|RB1\|PRDM16\|CBFA2T3\|NDUFA10\|LRPAP1\|AKT1\|HDAC4\|PIAS4\|JAK2\|THBS2\|CTDP1 |
| 31974 | Membrane-enclosed lumen | 1.01E-04 | 3.63E-02 | PACS2\|CTBP1\|NACC2\|BRF1\|FOXK1\|INTS1\|MTA1\|RB1\|PRDM16\|CBFA2T3\|NDUFA10\|LRPAP1\|AKT1\|HDAC4\|PIAS4\|JAK2\|THBS2\|CTDP1 |
| 70013 | Intracellular organelle lumen | 1.98E-04 | 4.18E-02 | PACS2\|CTBP1\|NACC2\|BRF1\|FOXK1\|INTS1\|MTA1\|RB1\|PRDM16\|CBFA2T3\|NDUFA10\|LRPAP1\|AKT1\|HDAC4\|PIAS4\|JAK2\|CTDP1 |
| 16585 | Chromatin remodelling complex | 2.17E-04 | 4.18E-02 | HDAC4\|MTA1\|RB1\|SMARCA4 |
| 33235 | Positive regulation of protein sumoylation | 2.33E-04 | 4.18E-02 | HDAC4\|PIAS4 |
| 33233 | Regulation of protein sumoylation | 2.33E-04 | 4.18E-02 | HDAC4\|PIAS4 |
| 51100 | Negative regulation of binding | 2.66E-04 | 4.24E-02 | HDAC4\|JAK2\|RB1\|LRPAP1 |
| 43467 | Regulation of generation of precursor metabolites and energy | 3.05E-04 | 4.38E-02 | AKT1\|HDAC4\|PRDM16 |
| 16564 | Transcription repressor activity | 3.67E-04 | 4.79E-02 | HDAC4\|SBNO2\|CTBP1\|NACC2\|PIAS4\|RB1\|PRDM16 |
