# Supplementary material for: Deoxyribonucleic acid methylation profiling of single human blastocysts by methylated CpG-island amplification coupled with CpG-island microarray
Source: Fertil Steril. 2015 Jun;103(6):1566–1571.e4. doi: 10.1016/j.fertnstert.2015.03.020 (PMC4449363; doi:10.1016/j.fertnstert.2015.03.020)
Supplement: Supplemental Table 5 [file mmc6.docx]

**Supplemental Table 5**

**Functional annotations of CGIs that are methylated in all 5 blastocysts as defined by DAVID TOOLS (The Database for Annotation, Visualization and Integrated Discovery).**

| **S.NO** | **KEGG pathways** | **Genes** |
| --- | --- | --- |
| 1 | Chronic myeloid leukaemia | C-terminal binding protein 1, SHC (Src homology 2 domain containing) transforming protein 2, retinoblastoma 1, v-akt murine thymoma viral oncogene homolog 1 |
| 2 | Focal adhesion | SHC, Collagen TypeVI alpha-1, thrombospondin 2, v-akt murine thymoma viral oncogene homolog 1 |
| 3 | Proto-oncogene | GNAS, MCF2, retinoblastoma 1, v-akt murine thymoma viral oncogene homolog |
| 4 | Glioma | SHC (Src homology 2 domain containing) transforming protein 2, retinoblastoma 1, v-akt murine thymoma viral oncogene homolog 1 |
| 5 | Small cell lung cancer | protein inhibitor of activated STAT 4, retinoblastoma 1, v-akt murine thymoma viral oncogene homolog 1 |
| 6 | ECM-receptor interaction | Agrin, Collagen type VI alpha 1, thrombospondin 2. |
