# Supplementary material for: Deoxyribonucleic acid methylation profiling of single human blastocysts by methylated CpG-island amplification coupled with CpG-island microarray
Source: Fertil Steril. 2015 Jun;103(6):1566–1571.e4. doi: 10.1016/j.fertnstert.2015.03.020 (PMC4449363; doi:10.1016/j.fertnstert.2015.03.020)
Supplement: Supplemental Table 6 [file mmc7.docx]

**Supplemental Table 6**

**Methylated CGIs that were identified by MCAM in human blastocysts that are within, or proximal to, known imprinted genes or predicted imprinted genes.**

| **Gene** | **Imprinting status** | **Chr.** | **Region methylated in embryos** | **CpG island** | **Number of embryos methylated** | **Known IG DMR at CGI?** |
| --- | --- | --- | --- | --- | --- | --- |
| *PRDM16* | predicted | 1 | 3102494- 3103389 | 55 | 4 | No* |
| *PRDM16* | predicted | 1 | 3111508- 3111935 | 25 | 2 | No* |
| *PRDM16* | predicted | 1 | 3327959- 3329467 | 107 | 3 | No* |
| *PRDM16* | predicted | 1 | 3341973-3342487 | 52 | 5 | No* |
| *OBSCN* | predicted | 1 | 228403224- 228403581 | 22 | 1 | No* |
| *OBSCN* | predicted | 1 | 228404338-228405085 | 23 | 1 | No* |
| *OBSCN* | predicted | 1 | 228473793-228474076 | 17 | 3 | No* |
| *OBSCN* | predicted | 1 | 228503542-228504117 | 94 | 4 | No* |
| *FAM50B* | imprinted | 6 | 3849242- 3849749 | 143 | 4 | Yes. DMR acquired during late preimplantation development. |
| *WDR27* | imprinted | 6 | 170059159-  170059624 | 25 | 4 | No |
| RHOBTB3 | imprinted | 5 | 95066815- 95067571 | 97 | 1 | Yes, placental-specific DMR |
| *MEST* | imprinted | 7 | 130132456- 130133192 | 177 | 3 | Yes |
| *DLGAP2* | imprinted | 8 | 1443833- 1444130 | 20 | 4 | No* |
| *DLGAP2* | imprinted | 8 | 1496686- 1497066 | 118 | 4 | No* |
| *TRAPPC9* | imprinted | 8 | 141108261-141110387 | 210 | 5 | Yes |
| *GLIS3* | imprinted | 9 | 4298501- 4299623 | 215 | 2 | Yes, placental-specific DMR |
| *GLIS3* | imprinted | 9 | 4118249- 4118669 | 64 | 3 | No |
| *AIFM2* | imprinted | 10 | 71892050- 71892405 | 67 | 4 | Yes, placental-specific DMR |
| *FAM196A/ DOCK1* | imprinted | 10 | 128993467- 128994033 | 172 | 4 | Yes, placental-specific DMR |
| *C10orf93* | predicted | 10 | 134733478- 134733849 | 79 | 5 | No* |
| *C10orf93* | predicted | 10 | 134729740- 134730056 | 17 | 4 | No* |
| *TRPM5* | Provisional data/disputed | 11 | 2435808- 2436701 | 106 | 3 | No* |
| *KCNQ1* | imprinted | 11 | 2593896- 2594282 | 20 | 3 | No |
| *KCNQ1OT1 (KvDMR1)* | imprinted | 11 | 2721216- 2721376 | 165 | 1 | Yes |
| *OSBPL5* | imprinted | 11 | 3141553- 3141922 | 16 | 2 | No |
| *NTM* | imprinted | 11 | 131780858- 131781287 | 121 | 2 | No, but placental-specific imprinting of *NTM* transcript has been reported. This CGI may be a putative placental-specific DMR. |
| *FBRSL1* | predicted | 12 | 133125634- 133126042 | 33 | 4 | No* |
| *FBRSL1* | predicted | 12 | 133073335- 133073588 | 23 | 2 | No* |
| *RB1* | imprinted | 13 | 48892830-48893870 | 85 | 5 | Yes |
| *FAM70B* | predicted | 13 | 114507521- 114508018 | 38 | 4 | No* |
| *FAM70B* | predicted | 13 | 114498004- 114498273 | 28 | 2 | No* |
| *HOXB3* | predicted | 17 | 46627711- 46628233 | 62 | 2 | No* |
| *ZNF331* | imprinted | 19 | 54058141- 54058313 | 83 | 4 | Yes |
| *DNMT1* | imprinted | 19 | 10304923- 10305280 | 89 | 4 | Yes, placental-specific DMR, also detected in preimplantation embryos |
| *L3MBTL1* | imprinted | 20 | 42143144- 42143539 | 35 | 2 | Yes |
| *GNAS XL* | imprinted | 20 | 57429008- 57429583 | 190 | 2 | Yes |
| *GNAS XL* | imprinted | 20 | 57430692-57431120 | 190 | 3 | Yes |
| NESP-AS/  GNAS-AS1 | imprinted | 20 | 57426722- 57427107 | 26 | 4 | Yes |
| NESP-AS/  GNAS-AS1 | imprinted | 20 | 57427603- 57428555 | 27 | 3 | Yes |
| *GNAS-A/B and*  *GNAS-1* | imprinted | 20 | 57465876-57466115 | 320 | 5 | Yes, but the imprinted DMR (EX 1A) is located at 57463265- 57465201 of the same CpG island. |

Methylated CGIs that were identified within known imprinted gene differentially imprinted regions (DMRs) are indicated. Information was compiled with reference to [www.geneimprint.com](http://www.geneimprint.com), the imprinted gene catalogue (<http://igc.otago.ac.nz/home.html>) and supplemental references (15-22) and may exclude some known tissue-specific DMRs. Chr.=chromosome number. No*= for the predicted imprinted genes, the location of the DMRs are not currently known.
